# A Development of Nucleic Chromatin Measurements as a New Prognostic Marker for Severe Chronic Heart Failure

**DOI:** 10.1371/journal.pone.0148209

**Published:** 2016-02-04

**Authors:** Machiko Kanzaki, Yoshihiro Asano, Hatsue Ishibashi-Ueda, Eiji Oiki, Tomoki Nishida, Hiroshi Asanuma, Hisakazu Kato, Toru Oka, Tomohito Ohtani, Osamu Tsukamoto, Shuichiro Higo, Hidetaka Kioka, Ken Matsuoka, Yoshiki Sawa, Issei Komuro, Masafumi Kitakaze, Seiji Takashima, Yasushi Sakata

**Affiliations:** 1 Department of Cardiovascular Medicine, Osaka University Graduate School of Medicine, Suita, Osaka, Japan; 2 Department of Pathology, National Cerebral and Cardiovascular Center, Suita, Osaka, Japan; 3 Center for Medical Research and Education, Osaka University Graduate School of Medicine, Suita, Osaka, Japan; 4 Research Center for Ultra-High Voltage Electron Microscopy, Osaka University, Osaka, Japan; 5 Department of Cardiovascular Science and Technology, Kyoto Prefectural University School of Medicine, Kyoto, Japan; 6 Department of Medical Biochemistry, Osaka University Graduate School of Medicine, Suita, Osaka, Japan; 7 Department of Cardiovascular Surgery, Osaka University Graduate School of Medicine, Suita, Osaka, Japan; 8 Department of Cardiovascular Medicine, Graduate School of Medicine, The University of Tokyo, Tokyo, Japan; 9 Department of Clinical Medicine and Development, National Cerebral and Cardiovascular Center, Suita, Osaka, Japan; University of Torino, ITALY

## Abstract

**Background:**

Accurate prediction of both mortality and morbidity is of significant importance, but it is challenging in patients with severe heart failure. It is especially difficult to detect the optimal time for implanting mechanical circulatory support devices in such patients. We aimed to analyze the morphometric ultrastructure of nuclear chromatin in cardiomyocytes by developing an original clinical histopathological method. Using this method, we developed a biomarker to predict poor outcome in patients with dilated cardiomyopathy (DCM).

**Methods and Results:**

As a part of their diagnostic evaluation, 171 patients underwent endomyocardial biopsy (EMB). Of these, 63 patients diagnosed with DCM were included in this study. We used electron microscopic imaging of cardiomyocyte nuclei and an automated image analysis software program to assess whether it was possible to detect discontinuity of the nuclear periphery. Twelve months after EMB, all patients with a discontinuous nuclear periphery (Group A, n = 11) died from heart failure or underwent left ventricular assist device (VAD) implantation. In contrast, in patients with a continuous nuclear periphery (Group N, n = 52) only 7 patients (13%) underwent VAD implantation and there were no deaths (p<0.01). We then evaluated chromatin particle density (Nuc-CS) and chromatin thickness in the nuclear periphery (Per-CS) in Group N patients; these new parameters were able to identify patients with poor prognosis.

**Conclusions:**

We developed novel morphometric methods based on cardiomyocyte nuclear chromatin that may provide pivotal information for early prediction of poor prognosis in patients with DCM.

## Introduction

A simple, accurate, and low-cost method to diagnose or prognosticate is always needed in the clinical setting. Over the past 2 decades, there have been significant advances in the treatment of heart failure [1–2], and many patients can now be rescued by recently integrated therapies. Physicians now have more opportunity to determine the indications for advanced therapy than ever before [3–5]. Since an early, appropriate decision to administer advanced medical therapy is essential for reducing the risk of cardiovascular events including death [6], we need a simple, accurate, and low-cost diagnostic tool to predict the prognosis of severe heart failure. Although prognostic markers for heart failure are currently supported by clinical practice guidelines [7], an effective single biomarker to determine whether left ventricular assist device (VAD) implantation will become necessary remains to be developed.

The degree of fibrosis and the morphology of cardiomyocytes can be assessed with light microscopy to determine the histological characteristics of cardiac tissue in patients with heart failure [8–10]. They are useful as qualitative markers; however, quantitative pathological measurements are not necessarily correlated with clinical outcomes [11–12]. In this study we focused on new ultrastructural findings with pathological examination of cardiac biopsy specimens. Recently, it has been shown that higher order structures in the cell nucleus change during development and differentiation, at times when gene expression is required [13–15]. This structural remodeling in the cell nucleus can be observed with electron microscopy as changes in high electron density areas called chromatin. Alterations in nuclear chromatin structure are associated with physiological changes such as neurological [16] or immunological function [17].

Based on our experimental findings of chromatin remodeling as an event upstream of pathological gene expression in cardiomyocytes [18], we focused on changes in cardiomyocyte nuclear chromatin structure in patients with dilated cardiomyopathy (DCM). We conducted a retrospective observational study to assess the ability of our original quantitation methods that analyze the ultrastructure of chromatin to identify DCM patients with severe heart failure at risk for poor outcomes, and to make an accurate and early determination of the necessity of a VAD to support the failing heart.

## Materials and Methods

### Patients

Between April 2009 and February 2013, 1121 patients were hospitalized for the treatment of heart failure at Osaka University Hospital. In the course of routine clinical diagnostic evaluation, cardiac biopsy was not performed in 950 of the 1121 patients in a non-biased manner because these patients have already been diagnosed with other diseases, such as congenital heart disease, ischemic heart disease, valvular heart disease, and other types of cardiomyopathy including secondary cardiomyopathy, or they did not consent to cardiac biopsy. As part of the diagnostic evaluation, 171 patients underwent endomyocardial biopsy (EMB). Of these, 63 patients with a low ejection fraction (≤35%) and New York Heart Association (NYHA) functional classification II–IV disease who were diagnosed with DCM were retrospectively reviewed. This study was approved by the research ethics committee of Osaka University Hospital and carried out in accordance with approved guidelines. Written informed consent was obtained from all patients before EMB.

### Patient Management and Follow-up

All baseline clinical measurements were made just prior to EMB. Patients underwent echocardiography, including color Doppler examination; coronary angiography; and routine laboratory testing. EMB was indicated for pathologic diagnosis only when chronic heart failure was stable and medically controlled. We followed patients for 12 months after EMB or the occurrence of a cardiac event, whichever came first. We collected follow-up data from the medical records for routine outpatient clinic visits.

In this study, we defined cardiac event as VAD implantation or death from heart failure. Indications for VAD implantation were based on Japan Circulation Society guidelines [19]. In short, they included irreversible end-stage heart failure with NYHA class IV disease by symptoms and imminent or already existing end-organ dysfunction despite optimal medical therapy, including dependent of inotropic agents. Hemodynamic criteria for VAD implantation included cardiac index ≤2.0 L/min/m^2^, systolic blood pressure ≤80 mmHg, and left or right atrium pressure ≥20 mmHg.

### Myocardial Tissue Analysis

For electron microscopic studies, we obtained more than 3 EMB samples from each patient. To obtain cardiac tissue in a minimally invasive manner, we performed cardiac biopsy of the right ventricle [11, 20–21]. We used contrast radiography during cardiac catheterization in order to obtained biopsy samples from different areas. Specimens were immediately fixed in 2.5% glutaraldehyde in 0.1 M phosphate buffer at 4°C and 1% osmium tetroxide, dehydrated with alcohol, and embedded in epoxy resin. We prepared semi-thin sections (1 μm thickness) and stained them with toluidine blue for optical microscopy. We also prepared thin sections (80 nm) and stained them with uranyl acetate and lead citrate. For each patient, each section was examined and photographed using a Hitachi H-7650 transmission electron microscope (TEM) (Hitachi, Tokyo, Japan). The entire area was photographed at 10,000x magnification. Electron microscopic images of the Z-disk in cardiomyocytes were obtained on an absolute scale. The images were fixed in grayscale, with 256 possible values, using image analysis software (WinROOF, Mitani, Tokyo, Japan). We adjusted the signal value of the Z-disk to the range of 155 to 165 in grayscale. Next, we calculate the signal of other organelle components on a relative scale to Z-disk. Therefore, the chromatin signal is a relative value corrected by the Z-disk value. This process was necessary to exclude brightness bias in the images. During image acquisition, we always verified that nuclei were from cardiomyocytes, not cardiac fibroblasts or endothelial cells. Each specimen had approximately 10 cardiomyocyte nuclei, on average. For optical microscopy, EMB specimens were stained with hematoxylin and eosin and Masson’s trichrome to measure the area of fibrosis and photographed with an Olympus DP71 digital camera (Olympus, Tokyo, Japan). For quantitative analysis, biopsy samples stained with Masson’s trichrome were digitally scanned at 20x magnification. The extent of fibrosis, which was stained blue, was automatically detected using WinROOF image analysis software. The mean percentage of interstitial fibrosis was calculated as the percentage of area with fibrosis divided by the entire area of the sample excluding the endocardium [22].

### Ultra High Voltage Electron Microscopy (ultra-HVEM) and Electron Tomography

We prepared samples by fixing and embedding. Specimens were immediately fixed in 2.5% glutaraldehyde in 0.1M phosphate buffer at 4°C and 1% osmium tetroxide, dehydrated with alcohol, and embedded in epoxy resin [23]. Samples embedded in resin were sectioned at 500 nm thickness and placed on a formvar-coated grid. After staining with 3% uranyl acetate in 70% methanol and lead citrate, gold particles (20 nm) were deposited on both surfaces of the section as fiducial markers and coated with carbon (JEE-420; JEOL, Tokyo, Japan). We stained 1 section per sample and obtained electron micrographs using an ultra-HVEM (H-3000, Hitachi) with an accelerating voltage of 1000 kV. A single axis tomography tilt series was recorded from −60° to +60° at 2° increments (61 slices per sample). The tilt series was aligned and reconstructed using weighted back-projection by the eTomo program in the IMOD software package [24]. For this study, we only focused on the intranuclear area.

### Classification of Group A and Group N Based on Quantitative Analysis

Quantitative measurements of chromatin ultrastructure seen under electron microscopy were made using the WinROOF image analysis software on a grayscale with 256 possible values. First, perinuclear condensed chromatin was isolated by extracting a dense spot with a value ranging from 146 to 256 to calculate the area of condensed chromatin. All the nuclei with discontinuous signals of perinuclear condensed chromatin by automatic detection were classified as Group A and those with continuous signals of perinuclear condensed chromatin were classified as Group N. During the process of chromatin assessment, we classified all nuclei in each specimen into either Group A or Group N.

### Calculation of the Chromatin Score and the Chromatin Surface Area Score in Group N

Two distinct morphometric parameters were calculated automatically by the WinROOF imaging analysis software.

Condensed chromatin was defined as having a value between 193 and 256 (i.e., the highest quartile of the 256 grayscale values). To normalize with respect to nuclear size, measurements of condensed chromatin were calculated as density per unit area and mean thickness. First, the ratio of the area of condensed chromatin to the area occupied by the perinuclear condensed chromatin was expressed as the nucleoplasmic chromatin score (Nuc-CS). We used the following formula to calculate Nuc-CS: Nuc-CS = [area of condensed chromatin with a value between 193 and 256] / [area contained by the perinuclear condensed chromatin except for the nucleolus] x 100. Second, we evaluated the area of perinuclear condensed chromatin per unit of the inner nuclear perimeter. The area of perinuclear condensed chromatin and the length of the inner nuclear perimeter were automatically measured with image analysis software. The average area of perinuclear condensed chromatin per unit of length was expressed as the perinuclear chromatin score (Per-CS), which was calculated using the following formula: Per-CS = [area of perinuclear condensed chromatin with a value between 193 to 256] / [length of the inner nuclear perimeter].

To determine the reproducibility and variability of Nuc-CS and Per-CS within a single heart, we performed these measurements for all observable nuclei in every patient sample for 6 randomly selected patients. In addition, interobserver agreement was analyzed by calculating the intraclass correlation coefficient for 30 randomly selected nuclei.

### Statistical analysis

Continuous variables are expressed as means ± SD or medians [interquartile range (IQR)]. Differences in clinical variables between the 2 groups were evaluated using Student’s t-test or the nonparametric Mann-Whitney U test, as appropriate. The chi-square test was used to compare categorical variables. A value of p<0.05 was considered to be statistically significant. Statistical analyses were performed using JMP10 software (SAS Institute, Cary, NC, USA).

## Results

### Electron Microscopic Analysis Strategy (Two-step Algorithm to Assess Chromatin Structure)

**Fig 1:**
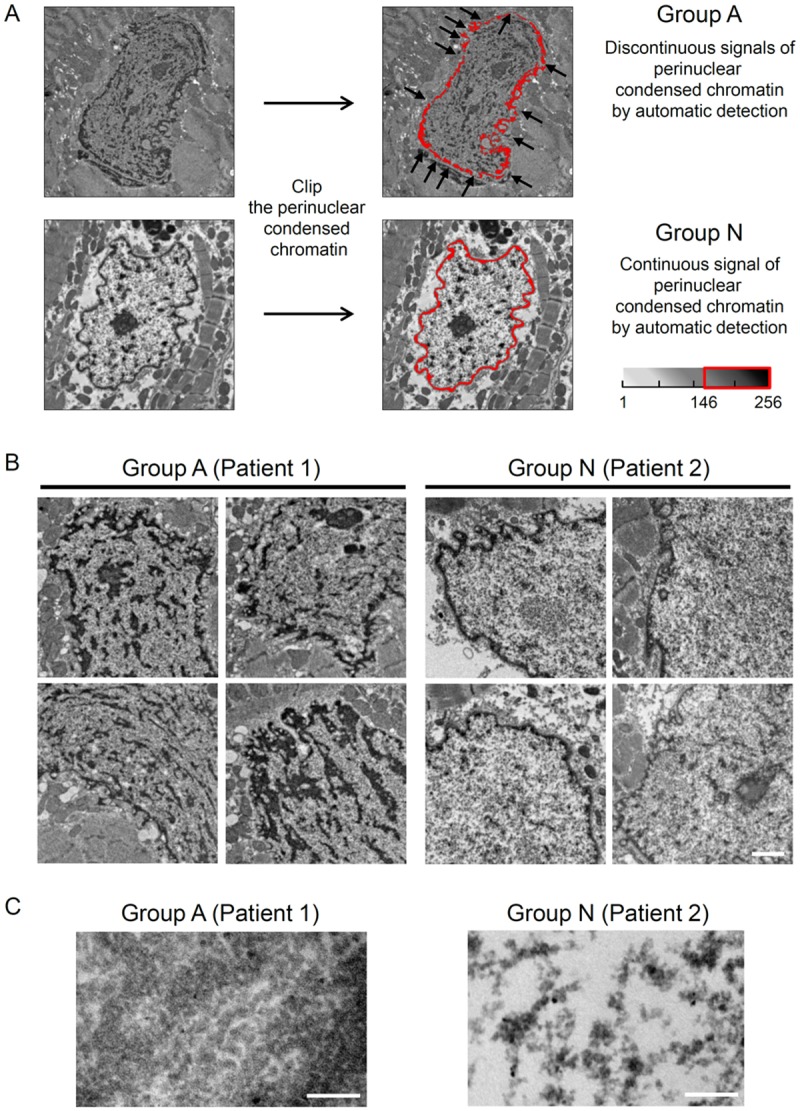
Classification of Groups A and N Based on Quantitative Analysis. (A) Representative original electron microscopic images before digital analysis are shown (10,000× magnification). The perinuclear condensed chromatin was isolated automatically using image analysis software. The area of perinuclear condensed chromatin, which is defined as the area with a grayscale value between 146 and 256, is outlined in red. Black arrows indicate areas of discontinuous nuclear periphery (discontinuous signals of perinuclear condensed chromatin by automatic detection). (B) Two types of nuclei, Group A and Group N, are shown. Representative conventional electron microscopic images (80 kV). Group A: These nuclei have discontinuous perinuclear condensed chromatin with irregular margins and unclear borders. There are homogeneous aggregations of particles in the nucleoplasm. Group N: These nuclei have continuous margins and clear borders. The granular particles in the nucleoplasm occasionally accumulate in spots, but do not aggregate closely. Scale bar: 1 μm. (C) High-power conventional electron microscopic (100 kV) images of chromatin structure in Patients 1 and 2, respectively. In Patient 1, the nucleus has an aggregated structure composed of poorly defined particles. In Patient 2, the nucleus has relatively sparse accumulations of particles less than 30 nm in size. Scale bar: 200 nm.

To quantitatively assess nuclear chromatin ultrastructure, we excluded patients whose nuclei were unable to be isolated because of the fuzziness of the nuclear border. As this process is defined by measurements made using the image analysis software, we could automatically categorize 2 types of nuclear ultrastructure based on the ability to isolate clear nuclear margins (Fig 1A). In Group A, the perinuclear condensed chromatin had some thin areas, so that the border signal was quite low and had the appearance of a signal defect (i.e., discontinuous signals of perinuclear condensed chromatin by automatic detection). On the contrary, Group N had a continuous signal. Based on this categorization we could find some striking differences in the morphology of the 2 groups. The nuclei in Group A also had irregular margins (Fig 1A (upper panel) and 1B (left panel)), and a homogeneous aggregation of particles in the nucleoplasm, most of which were fused together (Fig 1C (left panel)). Morphologically, the nuclei in Group N had continuous margins, clear borders (Fig 1A (lower panel) and 1B (right panel), and granular particles in the nucleoplasm that sometimes accumulated in spots, but most of the particle were isolated and not aggregated closely (Fig 1C (right panel)).

**Fig 2:**
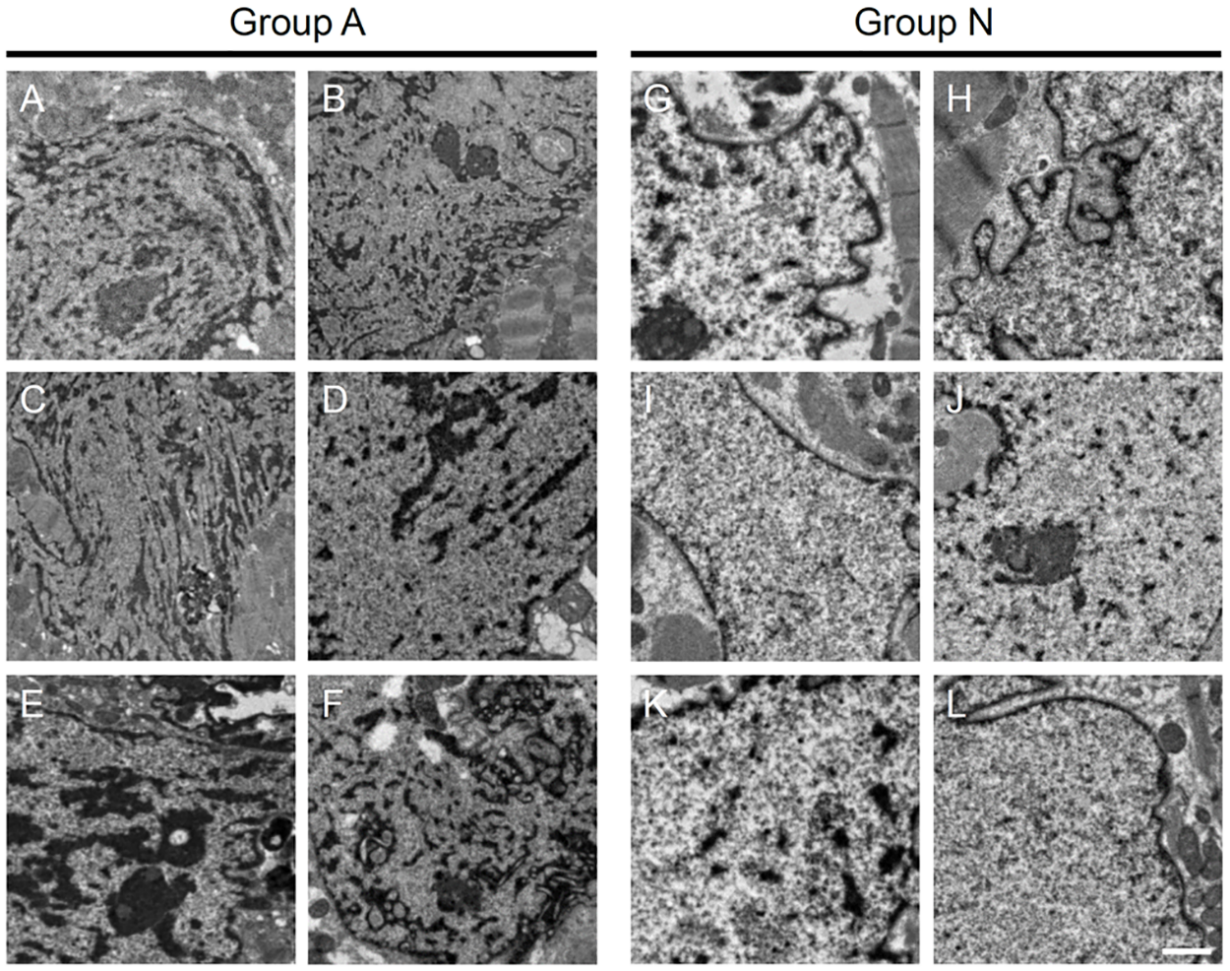
Representative Nuclei of 6 Patients from Each Group. Representative images are shown for each group. Six patients from Group A (A)–(F). Six patients from Group N: (G)–(L). Scale bar: 2 μm.

All sets of EMB samples from a single patient had similar morphological changes in all nuclei. Each patient had only one type of nuclear pattern. This indicated that these ultrastructural nuclear changes were homogeneous phenomena and not artifacts from sample preparation (Fig 2).

### Patients with Discontinuous and Heavily Condensed Chromatin Structure Have Poor Prognosis for Cardiac Events

Of the 63 patients analyzed in this study, there were 11 patients in Group A and 52 in Group N (Table 1). The patients in Group A were significantly younger than those in Group N (36.8 vs. 50.9 years, p = 0.006). Both left ventricular end-diastolic diameter (LVEDD) and left ventricular end-systolic diameter (LVESD) were significantly larger in Group A than in Group N (p = 0.02 and p = 0.007, respectively). We also measured other clinical parameters that have previously been reported to be correlated with the severity of heart failure [11–12, 21, 25–27], such as plasma brain natriuretic peptide (BNP) and the percentage of fibrosis in myocardial tissue. There were no significant differences in these clinical parameters between the 2 groups.

**Table 1:** Baseline Characteristic of Groups A and N.

	Overall (n = 63)	Group A (n = 11)	Group N (n = 52)	p value
**Demographic data**				
**Age (yrs)**	48.4 ± 15.9	36.8 ± 10.3	50.9 ± 12.8	0.006
**Male (%)**	51 (81.0)	9 (81.8)	42 (80.8)	0.93
**Clinical data**				
**NYHA functional class III and IV (%)**	38 (60.3)	8 (72.7)	30 (57.7)	0.41
**LVEF (%)**	24.6 ± 6.9	21.0 ± 7.9	25.8 ± 6.5	0.08
**LVEDD (mm)**	66.1 ± 11.5	74.5 ± 13.9	64.3 ± 10.2	0.02
**LVESD (mm)**	58.2 ± 12.9	69.1 ± 15.6	55.9 ± 11.2	0.007
**Hb (g/dL)**	13.1 ± 2.2	12.8 ± 2.1	13.2 ± 2.2	0.62
**Cr (mg/dL)**	1.2 ± 0.8	1.0 ± 0.3	1.2 ± 0.8	0.43
**T-bil (mg/dL)**	0.8 (0.3, 1.6)	1.0 (0.7,1.8)	0.7 (0.5, 1.5)	0.12
**BNP (pg/mL)**	269 (132, 528)	341 (229, 491)	259 (120, 572)	0.30
**% fibrosis (%)**	19.5 ± 13.1	23.7 ± 18.9	18.7 ± 11.6	0.61

Data are expressed as means ± SD, n (%), or medians (interquartile range).

NYHA, New York Heart Association; LVEF, left ventricular ejection fraction; LVEDD, left ventricular end-diastolic diameter; LVESD, left ventricular end-systolic diameter; Hb, hemoglobin; Cr, serum creatinine; T-Bil, total bilirubin; BNP, brain natriuretic peptide; % fibrosis, area of fibrosis within a specimen.

We followed the patients for 12 months after EMB. All 11 patients in Group A experienced a severe cardiac event (VAD implantation or cardiac death) (Table 2): 9 underwent VAD implantation and 2 died of progressive heart failure. In contrast, only 7 of 52 patients in Group N underwent VAD implantation and there were no deaths in this group. The rate of cardiac events was significantly higher in Group A compared to Group N (100% vs. 13%, p<0.01). Although the patients in Group A were younger and had more severe symptoms, larger left ventricular diameter, and a lower ejection fraction than those in Group N, these non-invasive clinical parameters could not predict their prognosis with the same degree of accuracy as nuclear morphology (Table 2). These results suggest that cardiomyocyte nuclei with discontinuous perinuclear condensed chromatin, irregular margins, unclear borders are associated with poor prognosis in patients with severe heart failure.

**Table 2:** Clinical Outcomes of Groups A and N.

	Group A (n = 11)	Group N (n = 52)
**VAD implantation**	9 (82%)	7 (13%)
**Cardiac death**	2 (18%)	0 (0%)
**No cardiac event**	0 (0%)	45 (87%)

Values are n (%). VAD, ventricular assist device; cardiac death, death due to heart failure.

### Establishment of Quantitative Morphometric Analysis of Intranuclear Ultrastructure

**Fig 3:**
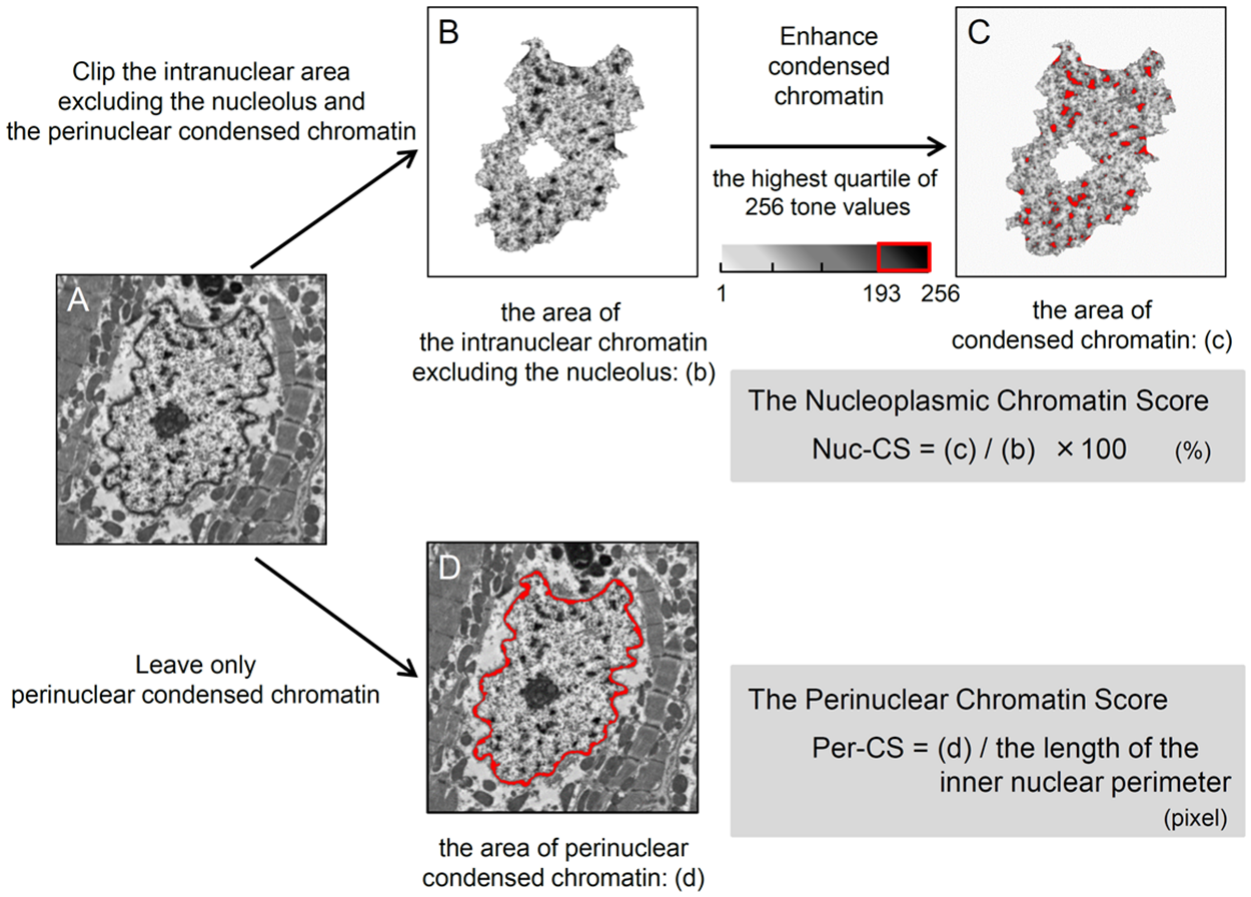
Protocol to Quantitatively Measure Chromatin Area in Group N. Our novel method is composed of the following steps. (A) Representative image prior to the availability of digital analysis (10,000× magnification). (B) The perinuclear condensed chromatin was isolated automatically by image analysis software. The area of the intranuclear chromatin excluding the nucleolus, shown as (b), was measured. (C) The area of condensed chromatin, defined as the area with a grayscale value between 193 and 256, was measured (red). The nucleoplasmic chromatin score (Nuc-CS) was defined as (c) / (b) ×100 (%). (D) The area of perinuclear condensed chromatin, defined as (d), and the length of the inner nuclear perimeter were automatically measured (outlined in red in (D)). The perinuclear chromatin score (Per-CS) was defined as (d) / the length of the inner nuclear perimeter (pixel).

We predicted extremely poor outcomes for Group A. However, patients in Group N still showed a broad range of heart failure outcomes, which conventional clinical parameters could not clearly predict with a high degree of accuracy. Based on the findings from our previous basic research experiments, we next aimed to classify patients in Group N by chromatin structure. In Group A, we could not clip out each nucleus from the whole image because its perinuclear condensed chromatin was discontinuous. In contrast, in Group N we could isolate each nucleus based on the clear border signal. The nuclei in Group N were evaluated using new quantitative parameters based on calculations of intranuclear chromatin structure isolated within the clear border signal (Fig 3).

**Fig 4:**
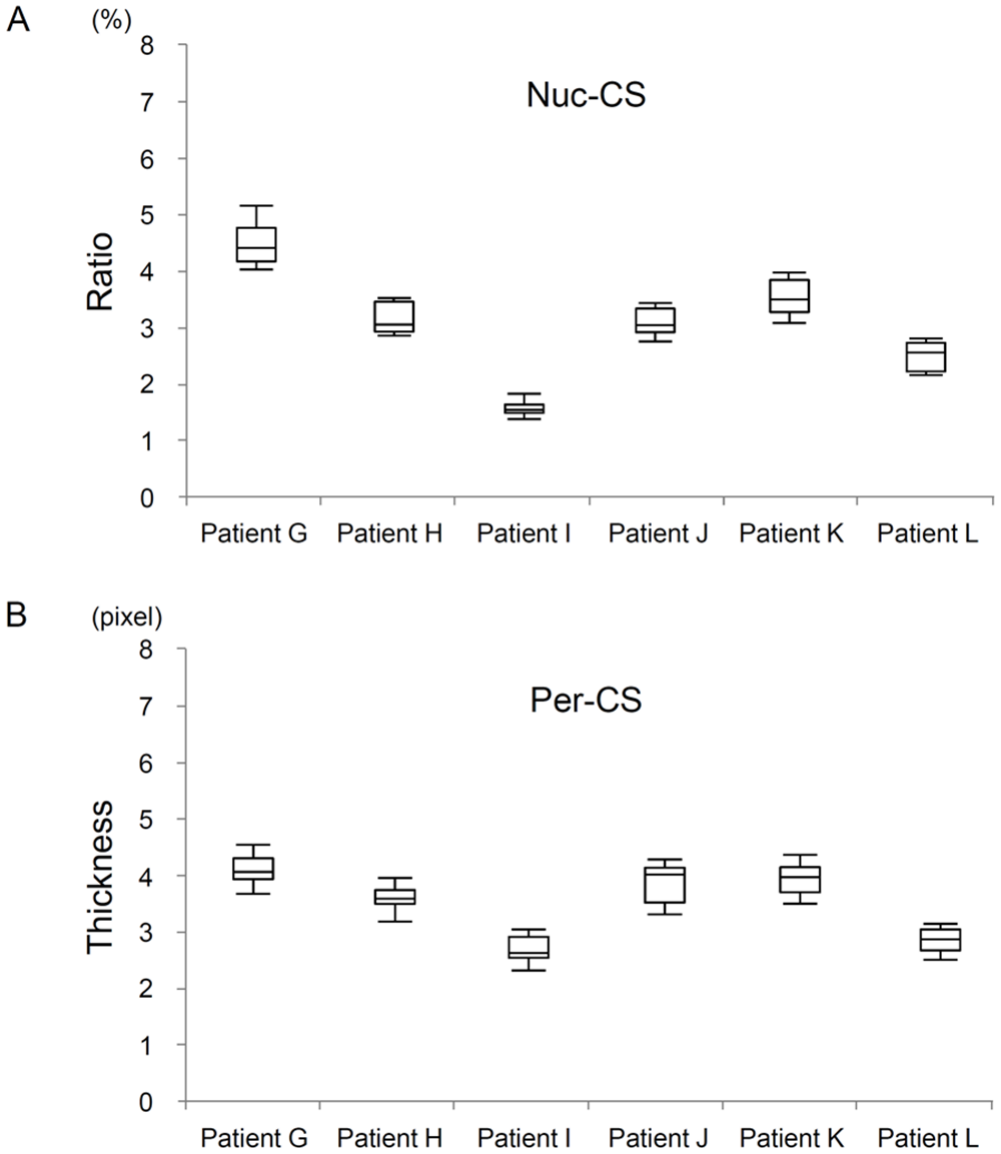
Variability in the Nucleoplasmic Chromatin Score (Nuc-CS) and the Perinuclear Chromatin Score (Per-CS). The variability in Nuc-CS and Per-CS in 6 Group N patients was measured. Six patients (Patients G—L) were randomly selected from Group N. In each patient, Nuc-CS was assessed for all observable nuclei; the variability is shown in the box plot. The top and bottom error bars represent the 90th and 10th percentiles, respectively. The top and bottom tiles of the box represent the 75th and 25th percentiles, respectively. The horizontal bar within the box represents the median.

These 2 parameters were originally intended to be used for quantitative classification of Group N. High Nuc-CS values represent either more accumulations or larger chromatin particles, referred to as the intranuclear heterochromatin area. On the other hand, a low Per-CS value represents a loss of perinuclear heterochromatin. Regarding the relationship between the 2 new measures, there was no significant variability between Nuc-CS and Per-CS within a single heart; they were highly reproducible (Fig 4). Thus, we could confirm the variability of our Nuc-CS and Per-CS measurements. The intraclass correlation coefficients for interobserver agreement for Nuc-CS and Per-CS were 0.997 (95% confidence interval (CI), 0.965 to 0.999, p<0.001) and 0.970 (95% CI, 0.918 to 0.999, p<0.001), respectively.

### Neither Nuc-CS nor Per-CS is Correlated with Conventional Clinical Prognostic Factors in Patients with Heart Failure

**Fig 5:**
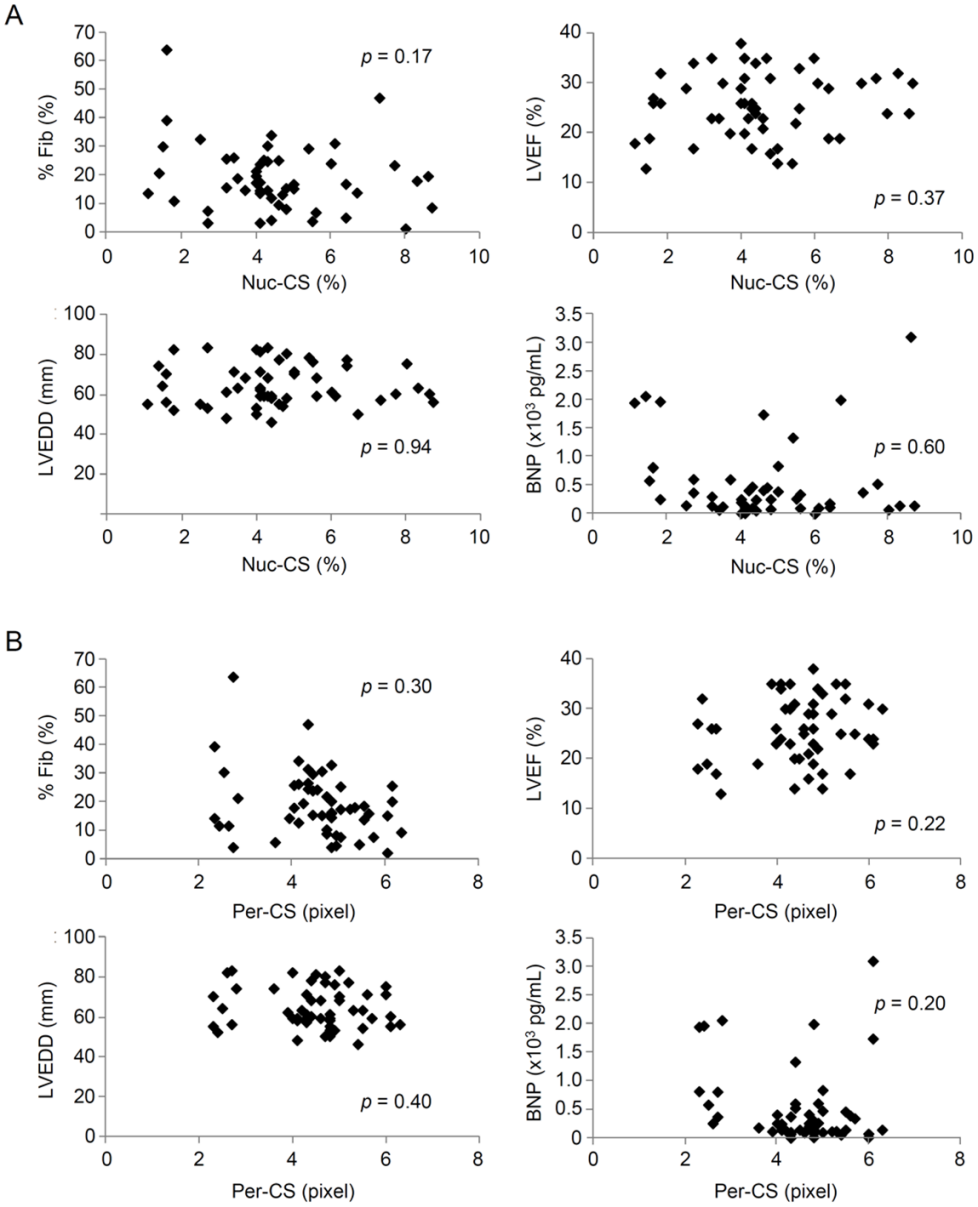
Relationship between the Nucleoplasmic Chromatin Score (Nuc-CS), Perinuclear Chromatin Score (Per-CS), and Clinical Parameters. Scatter plots of clinical parameters are shown. % Fib: area of fibrosis within the specimen, LVEF: left ventricular ejection fraction, LVEDD: left ventricular end-diastolic diameter, BNP: brain natriuretic peptide. p: correlation coefficient.

We measured the new morphometric values Nuc-CS and Per-CS for all samples and investigated the relationship between these measurements and conventional clinical prognostic factors in heart failure, such as area of fibrosis, LVEF, LVEDD, and plasma BNP levels. We found no significant relationship among them (Fig 5), indicating that Nuc-CS and Per-CS are independent of other clinical parameters. Although there are some reports referring a relationship between chromatin structure and age, there was no relationship between chromatin score and age among patients with Group N nuclei (S1 Fig).

### Both Nuc-CS and Per-CS Were Weakly Correlated with Each Other and Can Possibly Predict Cardiac Events

**Fig 6:**
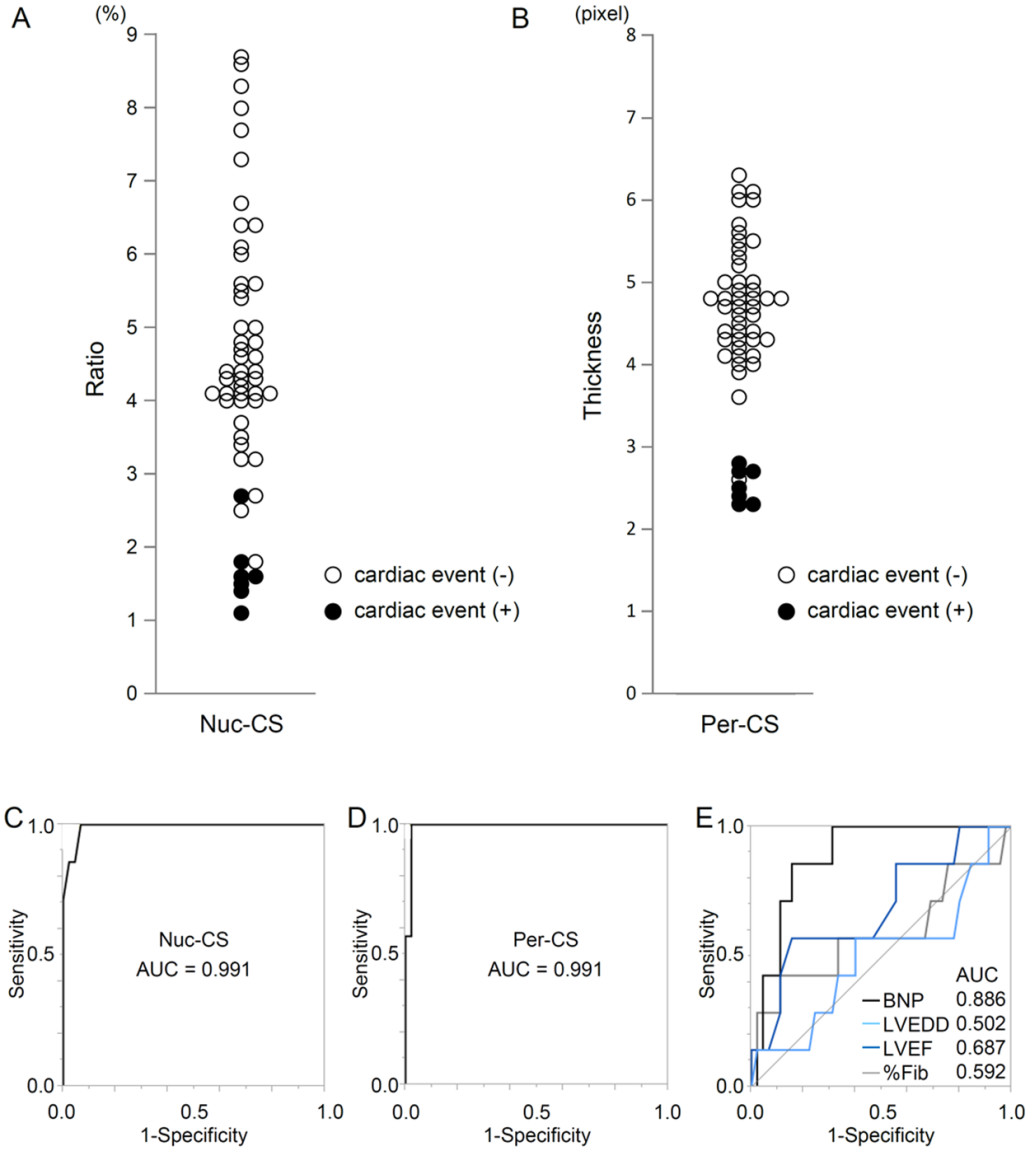
Distribution of Chromatin Score Values (Nuc-CS and Per-CS) and Cardiac Events. (A) Relationship between Nuc-CS and cardiac events. A white circle represents a patient who did not experience a cardiac event and a dark circle represents a patient who experienced a cardiac event. (B) Relationship between Per-CS and cardiac events. A white circle indicates a patient who did not experience a cardiac event and a dark circle represents a patient who experienced a cardiac event. (C) Receiver operating characteristic (ROC) curve analysis for Nuc-CS in predicting ventricular assist device (VAD) implantation 1 year after endomyocardial biopsy (EMB). (D) ROC curve analysis for Per-CS predicting VAD implantation 1 year after EMB. (E) ROC curve analysis for BNP (black), LVEDD (light blue), LVEF (dark blue), and %Fib (gray) in predicting VAD implantation 1 year after EMB. AUC, area under the curve; BNP, brain natriuretic peptide; LVEDD, left ventricular end-diastolic diameter; LVEF, left ventricular ejection fraction; %Fib, area of fibrosis within a specimen.

There was a weak correlation between Nuc-CS and Per-CS, suggesting some relationship between nuclear and peripheral chromatin formation in pathological states (S2 Fig). The relationship between cardiac events (i.e., VAD implantation or cardiac death) and Nuc-CS and Per-CS was then investigated. Nuc-CS (n = 52) ranged from 1.1% to 8.7% (Fig 6A). In Group N, 7 patients experienced cardiac events. Nuc-CS values of these 7 patients ranged from 1.1% to 2.7%. There was a distribution of Per-CS, with a range from 2.3 to 6.3 pixels (Fig 6B). Per-CS levels of the 7 patients who experienced cardiac events ranged from 2.3 to 2.8 pixels. Overall, patients with low Nuc-CS and low Per-CS values experienced significantly more cardiac events than those with higher values. Furthermore, receiver operating characteristic (ROC) curve analysis of Nuc-CS and Per-CS showed that they had significant discriminatory ability for predicting cardiac events (i.e., VAD implantation or cardiac death) 1 year after EMB (area under the curve (AUC_Nuc-CS_), 0.991; 95% CI, 0.9319 to 0.9987; AUC_Per_-_CS_, 0.991; 95% CI, 0.9211 to 0.9543) (Fig 6C and 6D). The AUC values for Nuc-CS and Per-CS were significantly greater than those for BNP, LVEF, LVEDD, and area of fibrosis (AUC_BNP_, 0.886; 95% CI, 0.7418 to 0.9543; AUC_LVEF_, 0.502; 95% CI, 0.257 to 0.745; AUC_LVEDD_, 0.687; 95% CI, 0.424 to 0.868; AUC_%Fib_, 0.592; 95% CI, 0.300 to 0.831) (Fig 6E).

## Discussion

To develop a biomarker to predict poor heart failure outcomes in DCM patients at an early stage, we designed this retrospective study to predict worsening of heart failure 1 year prior to cardiac events that required VAD implantation or resulted in death. Based on the findings from electron microscopic observations, we developed a novel non-conventional approach designed for morphometric quantitation of nuclear chromatin structure of the failing heart. To the best of our knowledge, this is the first quantitative evaluation of chromatin in clinical biopsy specimens.

Two different forms of chromatin were observed. One is the aggregated and discontinuous nuclear form (Group A), and the other is the non-aggregated and continuous nuclear form (Group N). In addition to the high-power conventional electron microscopic images shown in Fig 1C, we reconstructed 3-dimensional images of the same specimens using ultra-HVEM, which confirmed that these aggregations were composed almost entirely of small particles, less than 30 nm in size, which are believed to be folded chromatin structures (S1, S2, S3 and S4 Movies). These findings suggest that electron microscopy (Fig 1B), high-power conventional electron microscopy (100kV) (Fig 1C), and ultra-HVEM (S1, S2, S3 and S4 Movies) can distinguish between Group A and N chromatin microstructure.

It is not clear where Groups A and N fall into the sequence of chromatin alteration in the pathogenesis of heart failure. This approach revealed that the nuclei of Groups A and N do not exist concomitantly in the same specimen. One interpretation is that Groups A and N are not part of the same sequence for a certain disease; instead they have different etiologies. We think that potentially conflicting chromatin findings provide support for this hypothesis. More specifically, lower Nuc-CS and Per-CS scores indicate poor outcome for heart failure in Group N, but chromatin aggregation also indicates poor outcome for heart failure in Group A. Different mechanisms might be responsible for chromatin formation in Group N and chromatin aggregation in Group A, possibly due to different etiologies of heart failure. Based on the baseline characteristics in Table 1, the phenotype in Group A seems to be early-onset and progressive. There might be some differences in the etiology of DCM between Groups A and N. Genetic tests in the next study will hopefully resolve this question.

Group A patients were younger and had more dilated ventricles. It is highly plausible that progressive heart failure with onset at a younger age portends poor prognosis. However, there was some overlap in the distribution of age and ventricular size between Groups A and N. All young patients with large ventricles do not always have poor prognosis. Previous reports suggest that perinuclear heterochromatin thickness is inversely related to age [28–29]. However, in this study, Group A patients who are younger in average than Group N patients had thinner perinuclear heterochromatin. Epigenetic modifications might change transcriptional profiles based on aging. Many conceptual speculations and biochemical or immunohistological observations in certain experiments have already been reported elsewhere, but disease-related alteration of chromatin structure observed in histological samples has not been reported thus far. Our observations represent the first report.

**Fig 7:**
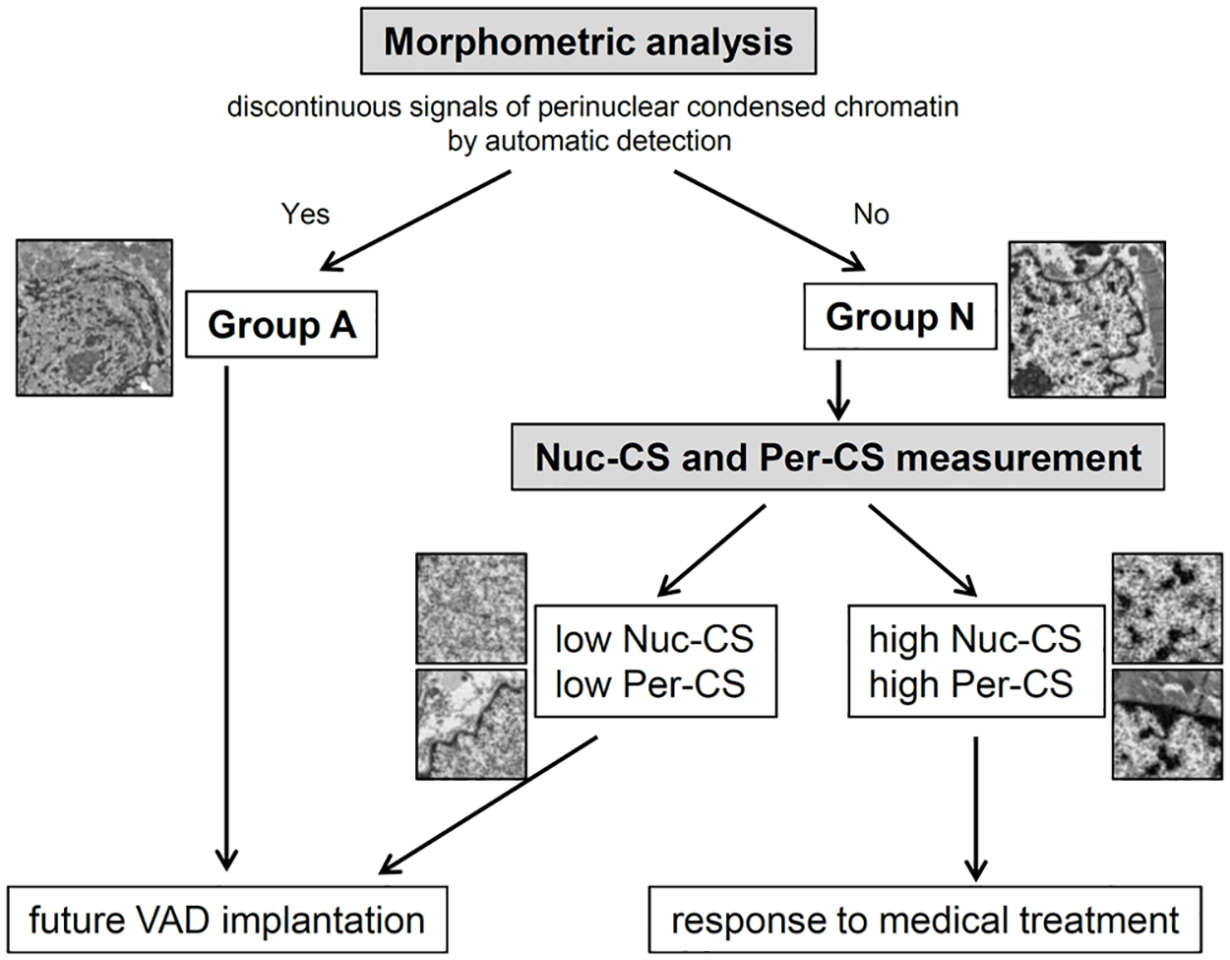
Algorithm for DCM Therapy Based on Myocardial Biopsy Parameters. The first step is morphometric analysis of cardiomyocyte nuclei. When chromatin in the nuclear periphery is discontinuous and there is a homogeneous aggregation of particles in the nucleoplasm, the patient is classified as Group A, which is associated with poor prognosis. When a patient does not have these characteristics, a second step, which involves additional quantitative analysis, is added. Based on the chromatin scores Nuc-CS and Per-CS, a treatment plan can be determined.

More importantly, chromatin discontinuity could clearly differentiate patients with good and poor outcomes. All patients in Group A experienced severe cardiac events within 12 months of EMB. Compared to Group A, Group N had more patients, with a broad range of prognosis. Therefore, a precise characterization of Group N was needed. We also developed 2 quantitative methods to further classify Group N, Nuc-CS and Per-CS. These two scores could accurately predict future need for VAD implantation in Group N. Taken together, we propose an algorithm for treating heart failure in patients with DCM (Fig 7).

For patients with severe heart failure, we have to assess whether VAD implantation and cardiac transplantation are indicated and what is the optimal timing [30]. There are several guidelines and criteria documents describing indications for such therapies [31]. Even though many different classification and scoring systems have been used to assess risk in patients undergoing VAD implantation, such methods still have weaknesses [32] because almost all of them are based on hemodynamic changes with coexisting severe symptomatic deterioration. For example, the INTERMACS profile [4] is a useful classification tool for predicting outcomes in patients with advanced heart failure receiving mechanical circulatory support from a VAD. However, this scale classifies such patients according to end-stage hemodynamic decompensation; these patients have often already experienced frequent remissions and exacerbations. Therefore, our goal is to find an effective biomarker that has predictive power for a cardiac event within 1 year, and use this marker for patients who have not yet experienced many hospital admissions for heart failure.

There are several reasons why we could effectively predict future deterioration with this new method. First, chromatin remodeling is considered an upstream event compared to macro-level tissue alterations, suggesting that it is strongly related to early disease status [13–17, 33]. Second, we only focused on cardiomyocyte nuclei, not on nuclei of other cell types. Since the cell population in cardiac tissue during end-stage heart failure becomes heterogeneous, it is not possible to eliminate the influence of fibroblasts and endothelial cells on conventional biomarkers. Third, nuclear chromatin structure can be rapidly preserved after cardiac biopsy. Biopsy samples do not undergo as much degradation as RNA, protein, and other fragile organelles such as mitochondria. Although a increased number of small mitochondria with disorganized cristae have been reported to be associated with the progression of heart failure in animal models, it is not always easy to judge changes in mitochondria under standardized conditions in the clinical setting.

As mentioned above, in this study we used novel morphometric methods to analyze cardiomyocytes in DCM. We have already started to apply these methods to human and murine thoracic aortic constriction (TAC) models of heart failure. We have already observed a relationship between morphological alterations in chromatin structure and progression of heart failure (data not shown). Preliminary evidence suggests that as heart failure worsens, chromatin structure changes; there is a decrease in the amount of intranuclear condensed chromatin and a thinning of peripheral chromatin. These findings suggest that chromatin morphometry can be used in the pathophysiological investigation of heart failure. To make the study protocol simpler, the utility of Nuc-CS relative to Per-CS can be determined in a future prospective study.

Our study has several limitations. First, biopsy samples were taken from the right ventricle. However, we have some data that suggest nuclear alteration of the right heart is comparable to that of the left. We have stored whole heart samples from cardiac transplant recipients. Multiple samples could be obtained from one heart from different locations (*e*.*g*. anterior, posterior, lateral, and septal wall, which are collected from both the endocardium and epicardium and both left and right ventricles). All the nuclei in EM samples from these specimens belonged to Group A. In animal models, nuclear chromatin structures differ, and the pathogenesis of chronic heart failure in animal models is different from that in humans, which is formed over years. Therefore, we cannot confirm changes in histopathology of chromatin alteration within a single heart.

Second, the calculation of Nuc-CS and Per-CS is based on the use of a relative grayscale measurement. In addition, chromatin density was evaluated as a dichotomized variable based on grayscale measurements. In order to simplify imaging analysis and to minimize the effect of variations in EM staining, we chose to dichotomize a continuous variable based on the highest quartile of grayscale values. After chromatin density alteration is better understood and technological advances in making such measurements occur, averaging a random number of grayscale measures to derive a mean chromatin density might strengthen the predictive value.

Third, we did not evaluate changes in chromatin structure over time in a single patient. To illustrate the point of no return with respect to chromatin ultrastructure, we have to assess the sequence of changes in nuclear phenotype for each group. For example, we need to investigate the sequence of changes in Group A versus Group N, and within Group N during with heart failure treatment. In order to verify the clinical utility of this method, a validation cohort with more patients and a longer follow-up period is needed.

We also have to compare this method to other noninvasive methods currently in clinical use, such as cardiopulmonary exercise testing, serial measurement of neurohormones, and response to therapies. Of course, we should include patients with other types of chronic heart failure such as other types of idiopathic cardiomyopathy, secondary cardiomyopathies, and mild to moderate heart failure (i.e., with a high ejection fraction (>35%) or NYHA class I disease). In the future, effective markers of reverse remodeling or the point of no return in advanced heart failure may guide decision-making regarding VAD implantation.

## Conclusions

We have developed novel methods to assess nuclear chromatin ultrastructure in cardiomyocytes. Using our methods, we could clearly identify patients with severe heart failure at risk for a poor outcome. Such evaluation of chromatin structure and morphology may serve as an early predictive biomarker, providing pivotal information on prognosis in patients with DCM.

## Supporting Information

S1 Fig Relationship between Chromatin Score and Age. Two-dimensional scatter plots of the nucleoplasmic chromatin score (Nuc-CS) and age, and the perinuclear chromatin score (Per-CS) and age. There is no correlation between either chromatin score and age.
(PDF)

S2 Fig Relationship between Nuc-CS and Per-CS. Two-dimensional scatter plot of the nucleoplasmic chromatin score (Nuc-CS) and the perinuclear chromatin score (Per-CS). There is weak correlation between Nuc-CS and Per-CS.
(PDF)

S1 Movie Three-dimensional tomographic images of the specimens in Group A using 1000 kV ultra- high voltage electron microscopy. (MOV)

S2 Movie Three-dimensional projections of the specimens in Group A using 1000 kV ultra- high voltage electron microscopy. (MOV)

S3 Movie Three-dimensional tomographical images of the specimens in Group N using 1000 kV ultra- high voltage electron microscopy. (MOV)

S4 Movie Three-dimensional projections of the specimens in Group N using 1000 kV ultra- high voltage electron microscopy. (MOV)

S1 Text Methods: Selection of electron microscopic images. (DOCX)
