# Supplementary material for: A Development of Nucleic Chromatin Measurements as a New Prognostic Marker for Severe Chronic Heart Failure
Source: PLoS One. 2016 Feb 4;11(2):e0148209. doi: 10.1371/journal.pone.0148209 (PMC4742272; doi:10.1371/journal.pone.0148209)
Supplement: S1 Fig — Two-dimensional scatter plots of the nucleoplasmic chromatin score (Nuc-CS) and age, and the perinuclear chromatin score (Per-CS) and age. There is no correlation between either chromatin score and age. (PDF) [file pone.0148209.s001.pdf]

S1 Fig

Kanzaki, et al.

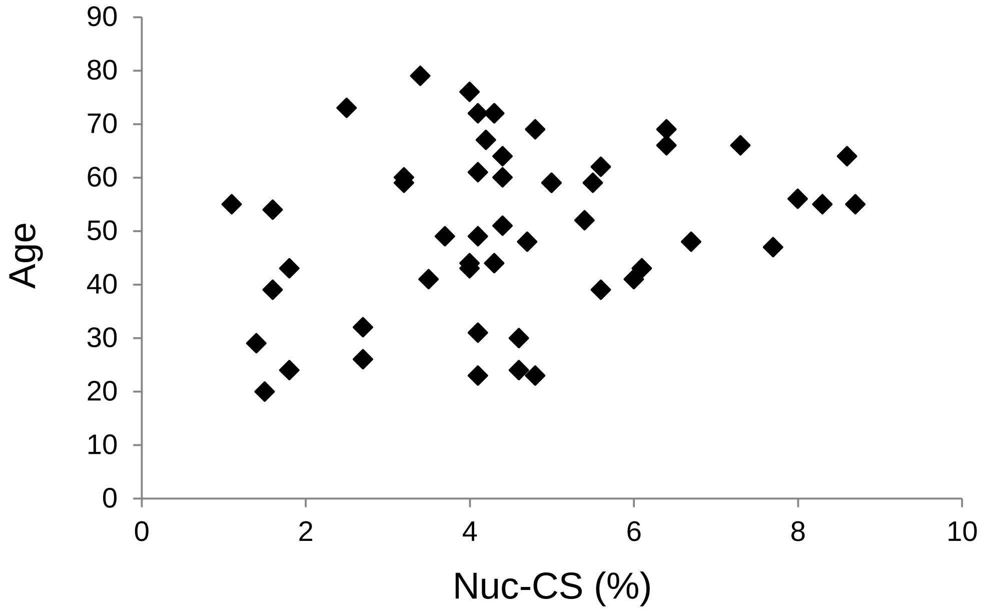

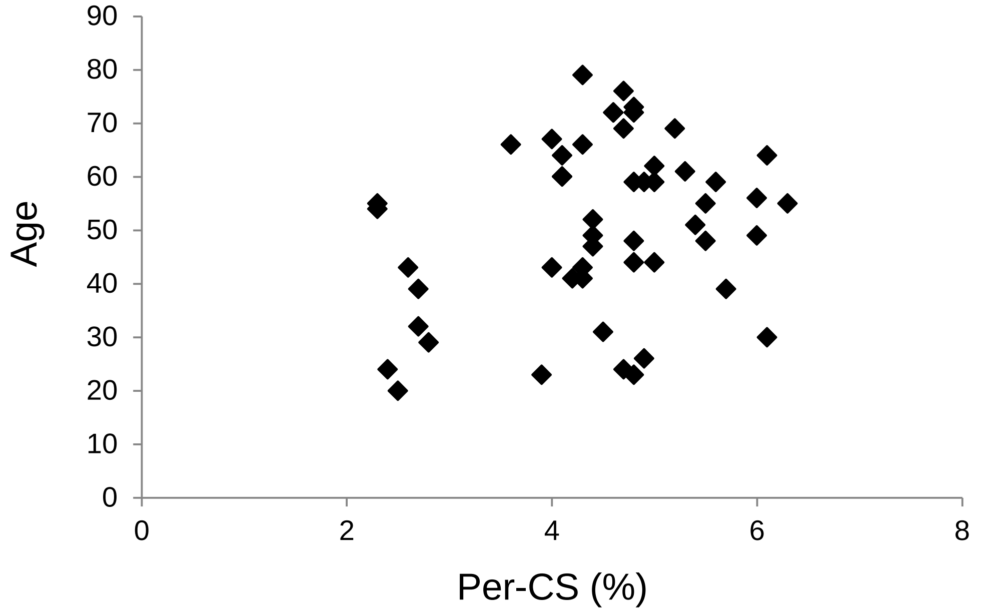

S1 Fig. Relationship between the Nucleoplasmic Chromatin Score (Nuc-CS), the Perinuclear Chromatin Score (Per-CS) and Age. Two-dimensional scatter plot of the nucleoplasmic chromatin score (Nuc-CS) and the perinuclear chromatin score (Per-CS) and age. There is no correlation between chromatin score and age.
