# Supplementary material for: A Development of Nucleic Chromatin Measurements as a New Prognostic Marker for Severe Chronic Heart Failure
Source: PLoS One. 2016 Feb 4;11(2):e0148209. doi: 10.1371/journal.pone.0148209 (PMC4742272; doi:10.1371/journal.pone.0148209)
Supplement: S2 Fig — Two-dimensional scatter plot of the nucleoplasmic chromatin score (Nuc-CS) and the perinuclear chromatin score (Per-CS). There is weak correlation between Nuc-CS and Per-CS. (PDF) [file pone.0148209.s002.pdf]

# S2 Fig

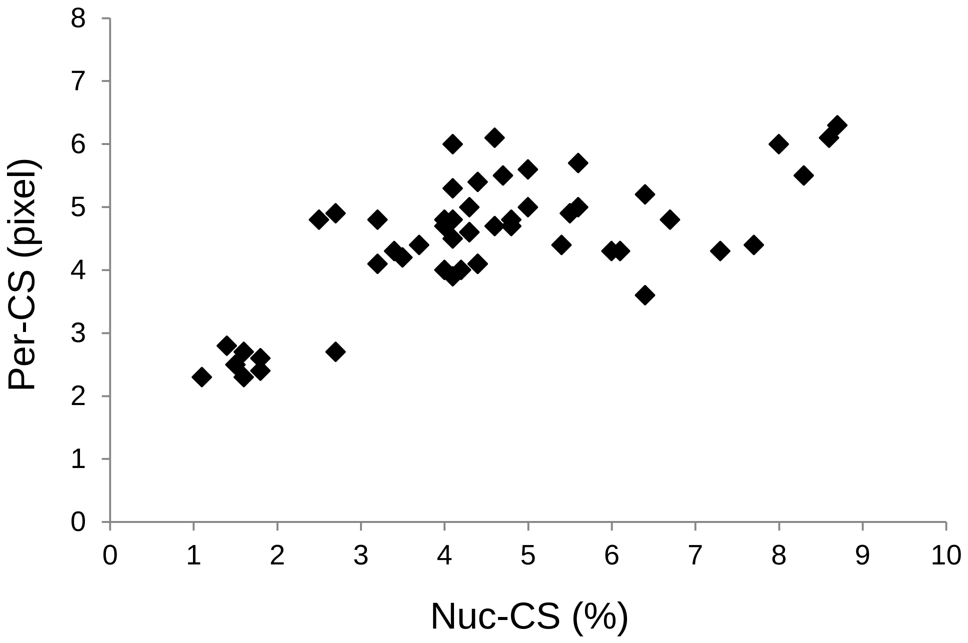


**S2 Fig. Relationship between Nuc-CS and Per-CS.**
Two-dimensional scatter plot of the nucleoplasmic chromatin score (Nuc-CS) and the perinuclear chromatin score (Per-CS). There is a weak correlation between Nuc-CS and Per-CS.
