# Supplementary material for: A Development of Nucleic Chromatin Measurements as a New Prognostic Marker for Severe Chronic Heart Failure
Source: PLoS One. 2016 Feb 4;11(2):e0148209. doi: 10.1371/journal.pone.0148209 (PMC4742272; doi:10.1371/journal.pone.0148209)
Supplement: S1 Text — (DOCX) [file pone.0148209.s007.docx]

**Supporting Information**

**S1 Text. Selection of electron microscopic images**

On average, each sample had approximately 10 cardiomyocyte nuclei. We had to exclude 2 to 3 non-quantifiable nuclei in each sample, which were ruptured during preparation (preparation artifact) of electron microscopic sections or located in a far corner of the section. Since they could not be analyzed by our imaging software, we defined such nuclei as non-quantifiable. In addition, in order to exclude samples in a poor state of preservation, we checked for structural disruption of mitochondrial cristae at 80,000x magnification. We have confirmed that there have been no cases with such disruption, thus far.
